# CoupleMDA: Metapath-Induced Structural-Semantic Coupling Network for miRNA-Disease Association Prediction

**DOI:** 10.3390/ijms26104948

**Published:** 2025-05-21

**Authors:** Zhuojian Li, Guanxing Chen, Guang Tan, Calvin Yu-Chian Chen

**Affiliations:** 1 School of Intelligent Systems Engineering, Shenzhen Campus of Sun Yat-sen University, Shenzhen 518107, China; lizhj39@mail2.sysu.edu.cn (Z.L.); chengx48@mail2.sysu.edu.cn (G.C.); 2 School of AI for Science, Peking University, Beijing 100871, China; 3 State Key Laboratory of Chemical Oncogenomics, Key Laboratory of Chemical Genomics, Peking University Shenzhen Graduate School, Shenzhen 518055, China; 4 Department of Medical Research, China Medical University Hospital, Taichung 40447, Taiwan

**Keywords:** heterogeneous graph, link prediction, miRNA-disease association, metapath

## Abstract

The prediction of microRNA-disease associations (MDAs) is crucial for understanding disease mechanisms and biomarker discovery. While graph neural networks have emerged as promising tools for MDA prediction, existing methods face critical limitations: (1) data leakage caused by improper use of Gaussian interaction profile (GIP) kernel similarity during feature construction, (2) self-validation loops in calculating miRNA functional similarity using known MDA data, and (3) information bottlenecks in conventional graph neural network (GNN) architectures that flatten heterogeneous relationships and employ over-simplified decoders. To address these challenges, we propose CoupleMDA, a metapath-guided heterogeneous graph learning framework coupling structural and semantic features. The model constructs a biological heterogeneous network using independent data sources to eliminate feature-target space coupling. Our framework implements a two-stage encoding strategy: (1) relational graph convolutional networks (RGCN) for pre-encoding and (2) metapath-guided semantic aggregation for secondary encoding. During decoding, common metapaths between node pairs structurally guide feature pooling, mitigating information bottlenecks. The comprehensive evaluation shows that CoupleMDA achieves a 2–5% performance improvement over the current state-of-the-art baseline methods in the heterogeneous graph link prediction task. Ablation studies confirm the necessity of each proposed component, while case analyses reveal the framework’s capability to recover cancer-related miRNA-disease associations through biologically interpretable metapaths.

## 1. Introduction

MicroRNAs (miRNAs), a class of approximately 22-nucleotide non-coding RNA molecules, regulate key biological processes including cell proliferation, differentiation, and apoptosis through targeted gene expression modulation [1,2,3]. Their mechanisms of action encompass mRNA degradation induction, translational repression, and epigenetic regulation [4]. Studies have demonstrated that aberrant miRNA expression correlates with various diseases: for instance, downregulation of miR-103/107 associates with amyloid-beta deposition in Alzheimer’s disease [5], while miR-105 shows elevated expression in breast cancer [6]. These findings underscore miRNAs’ potential as disease biomarkers [7].

Conventional wet-lab methods for identifying MDAs face critical bottlenecks, including prolonged experimental cycles and high per-test costs [8]. This has driven the development of bioinformatics approaches based on the functional association hypothesis, which posits that phenotypically similar diseases are associated with functionally related miRNAs. Capitalizing on the strong representation learning capabilities of deep learning in graph-structured data processing, GNN-based frameworks have emerged as cutting-edge solutions for discovering potential MDAs in complex biological networks [9,10]. Among these, heterogeneous graph attention network variants that learn topological features through node embedding and neighborhood aggregation have shown particular promise. These methods generally follow two operational phases: (1) data acquisition and construction of miRNA-disease similarity matrices and (2) graph structure construction with GNN-based neighbor feature aggregation, followed by decoding through feed-forward neural networks.

Current methodologies for constructing miRNA similarity matrices exhibit significant methodological homogeneity and operational risks of data leakage. First, the similarity matrices derived from miRNA sequences and gene interaction networks are inherently sparse, necessitating supplementation through either filling [11,12,13,14,15] or concatenation [16,17] with Gaussian interaction profile (GIP) kernel similarity matrices. Analysis of methodology descriptions and open-source implementations in references [11,12,13,14,15,16,17] reveals that these approaches typically compute GIP-based similarities using the complete known MDA matrix to generate embeddings, yet directly employ these embeddings in cross-validation without masking test samples. This operational flaw allows positive test samples to participate in similarity computation as prior knowledge. Ablation experiments in MHGTMDA [16] demonstrate that removing node embeddings generated from unmasked GIP data causes predictive AUC to plummet from 96% to 74%, confirming substantial performance inflation from data leakage. Notably, most open-source implementations store fixed miRNA similarity matrices for subsequent cross-validation, inherently introducing data leakage. Our experimental results indicate that under such operational protocols, even simple multilayer perceptrons (MLPs) achieve inflated performance metrics, which fail to reflect models’ true predictive capabilities. Although DAEMKL [18] and MGADAE [19] circumvent GIP-induced leakage through autoencoder architectures, the intrinsic parameter sensitivity of GIP methods and their heavy reliance on known network structures hinder models’ ability to capture nonlinear high-order relationships, frequently resulting in overfitting or underfitting.

Secondly, existing studies [11,12,13,14,18,19,20] predominantly utilize the miRNA functional similarity matrix MISIM developed by Wang et al. (2010) [21], where outdated data represents only a secondary limitation compared with its fundamental methodological flaws. The core issue lies in circular logic: MISIM calculates miRNA functional similarity using pre-existing MDA data, while subsequent research directly incorporates this similarity matrix as input features for MDA prediction models. This approach creates tight coupling between feature and target spaces, where similarity metrics derived from known associations are used to predict homologous relationships, essentially forming a self-validation loop. Such closed-loop designs may lead to over-optimistic model evaluation, significantly compromising real-world generalization capability. Although GRNMF [22] proposed inferring miRNA similarity through gene functional interaction networks (later adopted by MSGCL [23]) to avoid circular reasoning, its reliance on set-wise maximum similarity matching constitutes a manually designed heuristic algorithm capable of capturing only shallow linear relationships.

Consequently, constructing a robust miRNA similarity matrix faces inherent challenges due to deficient data sources: resultant matrices inevitably suffer from either (1) sparsity issues, (2) data leakage with self-validation loops, or (3) limited expressiveness and weak generalization. Similar fundamental constraints equally apply to disease similarity matrix construction.

Various molecular entities, such as proteins, genes, and lncRNAs, provide multi-level biological insights from complementary perspectives. Leveraging additional biological entities as mediators connecting miRNAs and diseases not only addresses data incompleteness in single-molecule analyses but also enhances predictive models with enriched input features [16,24]. HGCNMDA [24] introduces a gene layer to construct a miRNA-gene-disease heterogeneous network, learning feature embeddings through GNN models. MUSCLE [17] establishes three heterogeneous networks using drugs, mRNAs, and lncRNAs as mediators between miRNAs and diseases, with feature embeddings learned via graph attention neural networks. MHGTMDA [16] integrates eight biological entities (including lncRNAs, circRNAs, and proteins) to build a heterogeneous biological graph containing 16 association types, employing heterogeneous graph transformers for feature encoding. Although these methods construct relatively comprehensive graph data structures, they exhibit three critical limitations: (1) The models learn node representations by aggregating multi-hop neighborhood features based solely on edge-type relationships, failing to capture structural information between links through features like common neighbors; (2) The absence of metapath-guided neighbor aggregation prevents intuitive interpretation of captured semantic relationships; (3) Over-simplified decoders relying on basic Hadamard products inadequately process neighbor embeddings. This architectural design results in significant information loss during both encoding and decoding processes.

The prediction of MDAs inherently constitutes a link prediction problem in heterogeneous information networks, where model architecture design must synergistically integrate network heterogeneity and topological characteristics of link prediction, a critical factor enabling high-performance prediction in this study. Metapath-based heterogeneous GNN methods overcome the limitation of flattening heterogeneous relationships in conventional approaches through self-consistent semantic aggregation strategies that capture structural information within metapath subgraphs and fuse multi-semantic features [25,26]. A metapath, defined as an ordered sequence of node and edge types, effectively characterizes multi-level semantic relationships in heterogeneous graphs [27]. In biological entity heterogeneous graphs, numerous biologically meaningful metapaths exist. For instance, the “miRNA → lncRNA → mRNA → disease” metapath reflects the biological semantics where miRNAs regulate disease pathways through ceRNA mechanisms: miRNAs competitively bind with long non-coding RNAs (lncRNAs), thereby releasing their target mRNAs from suppression [28]. A concrete example involves MALAT1 sequestering the miR-200 family in lung cancer, which elevates ZEB1 mRNA expression and promotes epithelial–mesenchymal transition (EMT) [29,30]. Other representative metapaths include the following:

- miRNA → mRNA → disease: direct miRNA regulation of aberrant gene expression induces pathogenesis [31].
- miRNA → drug → disease: therapeutic drugs exert effects by modulating miRNA expression [32].
- miRNA → circRNA → mRNA → disease: the circRNA-miRNA-mRNA axis plays crucial regulatory roles in diseases [33].

These heterogeneous metapaths connecting node pairs of different types establish informational bridges between biological entities, enabling models to capture rich biological semantics while enhancing logical consistency and interpretability.

Recent advances in link prediction research have enhanced the representation of heterogeneous relationships by integrating structural features (SF) into GNNs. These SFs include common neighbors [34] and resource allocation [35], which enable joint semantic-topological modeling and improve heterogeneous relationship characterization [36]. However, current methods predominantly employ high-order encoders to process high-dimensional semantic and topological node features while relying on simplistic Hadamard products for node representation reconstruction in decoders. This imbalanced design creates an information bottleneck during encoding compression, ultimately leading to unavoidable information dissipation in the decoding phase [37,38].

**Figure 1:**
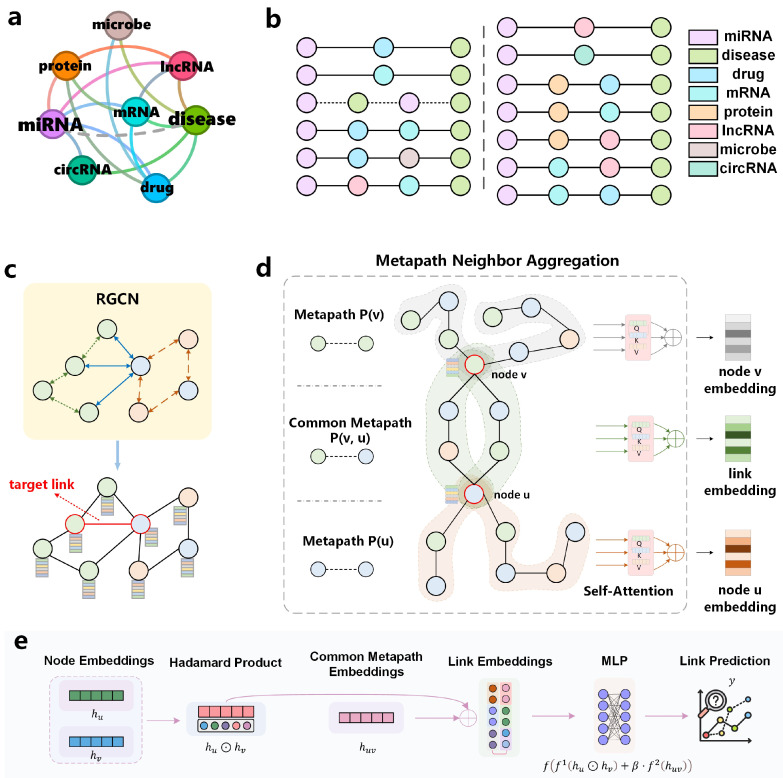
CoupleMDA model framework. (**a**) Heterogeneous graph linking relationships of biological entities; (**b**) 13 common metapaths discovered from the heterogeneous graph; (**c**) heterogeneous graph message passing neural network RGCN; (**d**) metapath neighbor aggregation module; (**e**) common metapath as a structure feature guided decoder.

To address these limitations, we propose CoupleMDA—a robust heterogeneous graph link prediction framework for biological entities that simultaneously captures structure-semantics coupled features of heterogeneous connections between miRNA and disease nodes. Specifically, we leverage the large-scale, heterogeneous graph of eight biological entities constructed by Zou [16] to mine metapaths and develop our model. Our framework autonomously learns the topological structure of heterogeneous graphs and infers target edge connections without over-reliance on heuristic algorithms with human-defined rules or biased prior knowledge. During data preprocessing, we strictly implement dataset partitioning and metapath generation to eliminate data leakage and self-validation loops. The proposed methodology operates through three phases, as shown in Figure 1:

(1) Primary encoding: employ RGCN to pre-encode all nodes in the original heterogeneous graph, establishing comprehensive preliminary topological relationships.
(2) Semantic augmentation: perform secondary encoding by fusing metapath semantic features on target edge nodes, enriching node embeddings with homogeneous neighborhood semantics.
(3) Structural decoding: guide structural decoding using common metapaths (CM) between target node pairs.

The intrinsic semantic information of metapaths combined with structural features from common metapaths enhances the framework’s coupling of graph structure and biological semantics. Guided by this design, we conduct extensive experiments to evaluate CoupleMDA’s superior performance against state-of-the-art baselines.

The contributions of our work are fourfold:

(1) We propose a novel framework for MDA prediction that automatically identifies diverse metapath weights (e.g., miRNA → lncRNA → mRNA → disease) from biological heterogeneous graphs, capturing cross-entity regulatory semantics to enhance biological interpretability.
(2) A new decoding mechanism incorporating common metapath structural features through attention-based dynamic weighting of multi-semantic pathways, enabling joint semantic-topological decoding to mitigate information bottlenecks.
(3) We abandon conventional similarity matrix construction methods (e.g., MISIM) that rely on known MDA data, instead utilizing non-associated data sources to eliminate feature-target space coupling and resolve self-validation loops.
(4) Extensive experiments demonstrate CoupleMDA’s significant outperformance over non-leakage state-of-the-art baselines across evaluation metrics, confirming its effectiveness and generalization capability in diverse scenarios.

## 2. Related Work

This section reviews two research directions: deep learning-based methods for MDA prediction and heterogeneous graph link prediction.

### 2.1. Deep Learning-Based MDA Prediction Methods

GNNs have emerged as the dominant framework for MDA prediction due to their capability to model relationships in biological networks. MHGTMDA [16] introduces a heterogeneous biological entity graph encompassing eight biomolecules, which comprehensively models indirect associations between miRNAs and diseases. HGCNMDA [24] constructs a miRNA-gene-disease heterogeneous network that subdivides node features into initial and inductive components to capture both direct and indirect associations. ReHoGCNES-MDA [11] adopts a homogeneous graph convolutional network with regular graph structures, where its random edge sampling strategy reduces training complexity while maintaining accuracy. ADPMDA [15] dynamically balances local and global node information by integrating an adaptive deep propagation GNN.

Multiple models focus on enhancing feature representation through multi-source data integration. DAEMKL [18] integrates miRNA and disease similarity networks via kernel learning, utilizing reconstruction errors from deep autoencoders to predict novel associations. MGADAE [19] combines multi-kernel learning with graph attention mechanisms, aggregating representations from multiple GCN layers to improve feature discriminability. NSAMDA [12] fuses miRNA sequence similarity with comprehensive similarity metrics, employing neighbor-selective graph attention networks to prioritize influential nodes. RFECV [14] implements a two-phase approach that uses deep attentive autoencoders with recursive feature elimination to selectively retain high-impact features in fused miRNA-disease similarity matrices. GCNPCA [20] combines GCN-derived topological features with PCA-based node attributes, achieving classification via random forests.

MSGCL [23] adopts multi-view self-supervised contrastive learning for MDA prediction, refining graph topologies by optimizing contrastive losses between anchor and learner views. MTLMDA [39] employs multi-task learning to jointly train miRNA-disease and gene-disease networks. CFNCM [13] generates association scores through collaborative filtering and classifies pairs using SVMs.

Notably, some models inadvertently introduce data leakage by mishandling GIP-generated miRNA similarity matrices. Others directly adopt MISIM for MDA prediction, entrenching self-validation loops. Even when avoiding these issues, such features capture only shallow linear relationships.

### 2.2. Heterogeneous Graph Link Prediction

Heterogeneous graph link prediction aims to infer potential or missing relationships between entities in heterogeneous networks. Metapaths have been widely adopted as essential tools for capturing semantic information in heterogeneous networks. Paths2Pair [40] proposes an entity pair screening strategy based on metapaths, which enhances prediction efficiency through content information aggregation. MAGNN [26] develops an intra- and inter-metapath aggregation framework that combines intermediate semantic nodes with multi-path information on node content transformation, improving link prediction performance. EMAA [41] introduces a bidirectional biased random walk algorithm that integrates RNNs and attention mechanisms to explore explicit and implicit metapath semantics. MHGNN [42] captures high-order dependencies in biological heterogeneous graphs through metapath aggregation and drug–target pairs. MV-HRE [43] jointly utilizes metapath views, community views, and subgraph views, aggregating contextual information via relation-aware attention mechanisms.

MLAN [44] designs a meta-learning-based adaptive network that enhances generalization by transferring shared knowledge through historical link-type community subtasks. MTTM [45] employs an adversarial learning framework where generative predictors and discriminative classifiers compete to learn transferable cross-link feature representations. NOH [38] proposes a heterogeneous hypergraph neural network that integrates low-order neighborhood overlaps with high-order group interactions. HeteHG-VAE [46] maps heterogeneous information networks into hypergraphs, learning latent node and hyperedge representations through Bayesian deep generative frameworks, while modeling multi-level relationships via hyperedge attention modules. THGNN [47] develops a topic-aware heterogeneous graph neural network that extracts multi-topic semantics from textual content through alternating aggregation mechanisms.

However, these models generally suffer from imbalanced encoder–decoder designs that inevitably cause information dissipation during decoding. NCNC [48] addresses this limitation by proposing a novel MPNN(Message Passing Neural Network)-then-SF architecture with neural common neighbors, which guides graph representation learning through structural features to achieve high performance. This approach effectively enhances model generalization by mitigating common neighbor decay and distribution shifts caused by graph incompleteness.

## 3. Preliminary

In this section, we present key concepts and formal definitions related to heterogeneous graph link prediction.

**Definition** **1** (Heterogeneous Graph)**.** *A heterogeneous graph is formally defined as G=(V,E,T,R,τ:V→T,ϕ:E→R), where $V=\{v_{1},v_{2},\ldots ,v_{n}\}$ is the set of nodes, each belonging to a predefined node type set T. $E=\{e_{1},e_{2},\ldots ,e_{n}\}\subseteq V\times V$ is the set of edges, each belonging to a predefined edge type set R. τ(v)∈T is the node type mapping function, and ϕ(e)∈R is the edge type mapping function. This definition characterizes the diversity of nodes and edges in heterogeneous graphs.*

**Definition** **2** (Metapath)**.** *A metapath P is formally defined as a relation pattern on a heterogeneous graph: $P=T_{1}\overset{R_{1}}{⟶}T_{2}\overset{R_{2}}{⟶}...\overset{R_{k}}{⟶}T_{k+1}$, where $T_{1},T_{2},\ldots ,T_{k+1}\in T$ is a sequence of node types in the heterogeneous graph, and $R_{1},R_{2},\ldots ,R_{k+1}\in R$ is a sequence of edge types connecting adjacent node types. Metapaths reflect complex semantic associations between different types of nodes in heterogeneous graphs.*

**Definition** **3** (Link Prediction)**.** *The goal of link prediction is to predict edges that do not yet exist in the graph. Given a pair of nodes $\left(v_{i},v_{j}\right)$, we aim to predict whether an edge will form between them. We formalize this task as ${\hat{y}}_{ij}=f\left(v_{i},v_{j},T,R\right)$, where ${\hat{y}}_{ij}$ is the predicted value of whether an edge exists between nodes $v_{i}$ and $v_{j}$. This definition transforms the link prediction problem into a binary classification task based on node and relation types.*

## 4. Results

This section evaluates the effectiveness of CoupleMDA through extensive experiments. Specifically, we first introduce the experimental setup, including datasets, evaluation metrics, and baseline methods. Subsequently, we provide a detailed analysis of the experimental results and validate the contributions of individual model components through ablation studies.

### 4.1. Datasets

To comprehensively evaluate the model’s performance on heterogeneous graph link prediction tasks, we first tested it on three representative public heterogeneous graph datasets. Then, we applied CoupleMDA to a previously constructed biological heterogeneous information network for MDA prediction testing.

The three heterogeneous graph datasets mentioned above are DBLP, LastFM, and Amazon. These datasets all use versions organized by HGB [31]. The specific details of each dataset are as follows:

- DBLP: This dataset is a subset of an academic network, which, after cleaning, contains rich academic elements: it covers 4057 researchers and their 14,328 published academic papers, along with 7723 professional terms and 20 types of academic publishing institutions.
- LastFM: This is a music social network dataset used to track and analyze users’ music listening behaviors. It continuously collects cross-platform music playback behavior feature data from users. After information cleaning and standardization, the constructed graph dataset covers multiple dimensions: 1892 listeners, 17,632 music creator accounts, and 1088 associated genre tags.
- Amazon: This dataset comes from user behavior data on an e-commerce platform. It includes product attributes and co-browsing and co-purchasing links between products. Product attributes include price, sales ranking, brand, and category.

We constructed our biological heterogeneous information network based on the dataset from Zou [16]. The training data were derived from the MDA database in HMDDv3.2, from which we selected 901 miRNAs and 877 diseases to build an adjacency matrix. To enhance the dataset, we integrated updated MDA data from HMDDv4.0 [49] and merged diseases with semantic similarities from the HMDD database. We added 75% of the MDAs as the training set to the graph. Other biological entities incorporated include 3348 protein nodes from the STRING database [50], 2633 lncRNA nodes from NONCODEV5 [51], 421 circRNA nodes from CircBase [52], 1319 drug nodes from DrugBank [53], 3024 mRNA nodes from the NCBI database [54], and 100 microbial nodes from the NIH Medical Subject Headings (MeSH) database [55]. Finally, an equivalent number of non-MDAs were randomly selected as negative controls. The data on associations between biological entities were collected from existing public databases and compiled by Zou et al. [16]. The relevant database sources are listed in Table 1.

### 4.2. Comparison Methods

To comprehensively evaluate the performance of CoupleMDA, we selected representative heterogeneous graph neural network models and MDA prediction models as baselines. These baseline models can be categorized into three classes:

#### 4.2.1. General Heterogeneous Graph Neural Networks

- RGCN [69]: This model extends traditional graph convolutional network (GCN) to heterogeneous graph scenarios by performing convolutional operations separately on different types of edges, followed by weighted aggregation. Its core innovation lies in introducing specific weight matrices for each relation type, effectively handling multi-type edge relationships in heterogeneous graphs.
- HGT [70]: This model introduces a heterogeneous graph transformer architecture, processing different types of nodes and edges through a heterogeneous attention mechanism. Its key feature is leveraging multi-type edge information as weights for message passing and capturing long-range dependencies via a metapath-guided transformer structure.
- GATNE [71]: This method learns three distinct embeddings for each node: general embeddings, edge-type-specific embeddings, and attribute-enhanced embeddings. By fusing these embeddings, the model captures both global node characteristics and relation-specific features.
- HetGNN [72]: This model employs type-aware random walk strategies to sample heterogeneous neighbors and processes heterogeneous information through a two-layer aggregation mechanism. The first layer aggregates nodes of the same type, while the second layer fuses features across different types, followed by end-to-end optimization of node representations.
- Simple-HGN [73]: This model enhances graph attention networks (GAT) performance by introducing three key components: learnable type embeddings, residual connections, and L2 normalization of output embeddings. These simple yet effective modifications significantly improve performance on heterogeneous graphs.
- HAN [74]: This model designs hierarchical attention mechanisms, including node-level attention and semantic-level attention. Node-level attention aggregates information within individual metapaths, whereas semantic-level attention integrates semantic information from multiple metapaths.
- MAGNN [26]: The primary innovation of this model lies in preserving and leveraging intermediate node information in metapaths. By designing a metapath instance encoder, the model captures complete path semantics rather than relying solely on path endpoints.

#### 4.2.2. MDA Prediction Models

- HGCNMDA [24]: This method introduces a gene layer to construct a miRNA-gene-disease heterogeneous network. It employs a multi-relational GCN to encode representations for given miRNAs, diseases, or genes, integrating neighbor representations according to distinct relation types.
- MHGTMDA [16]: This approach constructs a heterogeneous biological entity graph containing eight biomolecule types to comprehensively model indirect associations between miRNAs and diseases. The model processes diverse node and edge types through a heterogeneous attention mechanism, utilizing multi-type edge information as message-passing weights and capturing long-range dependencies via a metapath-guided transformer architecture.

#### 4.2.3. Link Prediction Models

- NCNC [48]: This model proposes an MPNN-then-SF framework and introduces the concept of neural common neighbors. By combining message passing with structural features, it significantly improves link prediction performance. Additionally, the model incorporates mechanisms to address graph incompleteness, thereby enhancing prediction generalizability.

### 4.3. Experimental Setup

For data partitioning, we randomly divided all existing edges (positive samples) in the dataset into training, validation, and test sets with a ratio of 75:10:15. Negative samples were sampled from node pairs that did not have any existing edges in the graph at a 1:1 ratio. To prevent information leakage, the training graph only contained positive edges from the training set, while positive edges in the validation and test sets were removed from the graph during training. We use the results on the test set as the final criterion for evaluating the performance of the model.

Model performance was evaluated using the receiver operating characteristic area under curve (ROC-AUC) and average precision (AP) scores. Additional core classification metrics included precision, recall, and F1 score:

(1) $$\mathrm{Precision}\;\left(P\right)=\frac{\mathrm{TP}}{\mathrm{TP}+\mathrm{FP}},$$

(2) $$\mathrm{Recall}\;\left(R\right)=\frac{\mathrm{TP}}{\mathrm{TP}+\mathrm{FN}},$$

(3) $$F1\;\mathrm{score}\;=\frac{2\cdot P\cdot R}{P+R},$$

where TP denotes true positives, FP represents false positives, and FN indicates false negatives.

The AUC metric measures the probability that a classifier ranks random positive samples higher than random negative samples:

(4) $$\mathrm{AUC}=\frac{\sum_{i=1}^{M}\sum_{j=1}^{N}\left[I\left(s_{i}>t_{j}\right)+0.5\cdot I\left(s_{i}=t_{j}\right)\right]}{M\cdot N},$$

where *M* and *N* denote the numbers of positive and negative samples, $s_{i}$ represents the prediction score of the *i*-th positive sample, $t_{j}$ indicates the prediction score of the *j*-th negative sample, and I(·) is an indicator function that returns 1 when the condition is satisfied. The AP metric approximates the area under the precision–recall curve using the trapezoidal rule:

(5) $$\mathrm{AP}=\sum_{k=1}^{n-1}\left(R_{k+1}-R_{k}\right)\cdot \frac{P_{k}+P_{k+1}}{2},$$

where $R_{k}$ and $P_{k}$ represent the recall and precision at the *k*-th threshold.

For experimental configuration, all models used the same five random seeds for dataset partitioning and training, with five independent experimental repetitions. Each different random seed will divide different training and validation sets, but the fixed test set remains invisible to the model before final evaluation. We employed the Adam optimizer with weight decay for parameter optimization and tuned hyperparameters based on validation set performance. All baseline implementations were adapted from their official codebases, with modifications made to their data loading interfaces and downstream decoders. Experiments were conducted on an NVIDIA RTX 4090D GPU with 24 GB of memory.

### 4.4. Performance Analysis

This section first presents the experimental results of different models on heterogeneous graph link prediction tasks using the DBLP, LastFM, and Amazon datasets to demonstrate the superiority of the CoupleMDA model on general datasets. It then shows the experimental results of different models on MDA prediction tasks using the previously constructed biological heterogeneous information network to verify the robustness of CoupleMDA on specialized datasets.

The experimental results for link prediction on general heterogeneous graph datasets are shown in Table 2. Experiments demonstrate that CoupleMDA consistently outperforms other baselines across different datasets. Compared with the best baseline (NCNC), CoupleMDA achieves a performance improvement of approximately 2–5%, indicating that co-metapaths, as structural information guiding GNN pooling, can maximize information capture. From the performance of different types of methods, metapath-based approaches generally outperform relation-based methods, suggesting that metapaths better capture semantic information in heterogeneous graphs. Further analysis reveals that CoupleMDA performs exceptionally well on real-world datasets with well-structured graph data. This is because, for graphs with fewer isolated edges, the more complete the neighborhood connections of the target edge, the better the model can leverage the coupled advantages of structure and semantics to deliver more accurate predictions.

**Figure 2:**
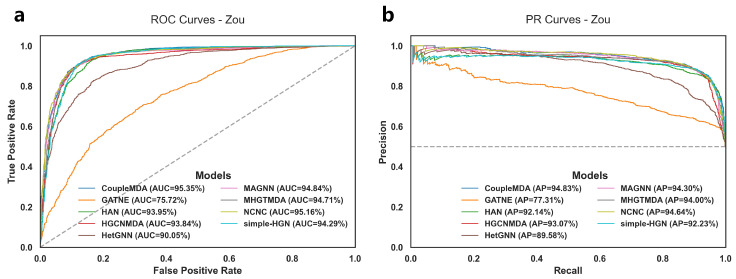
Experimental results of ROC and PRC curves for the MDA link prediction task on Zou’s dataset using CoupleMDA and baseline models: (**a**) ROC curve with dashed line y = x indicating random guessing; (**b**) PRC curve with dashed line y = 0.5 showing chance-level precision under balanced classes.

The experimental results for MDA link prediction on the test set are shown in Table 3 and Figure 2. The performance of different models on the training set and validation set, as well as detailed results on the test set, are provided in Appendix A.1. Specifically, Table A1 records the average metrics of five experiments for different baseline models on the training, validation, and test sets of the Zou dataset. Table A2, Table A3, Table A4, Table A5, Table A6, Table A7, Table A8, Table A9, Table A10 each record the TP, TN, FP, and FN values for each baseline model on the test set in each experiment. Notably, HGCNMDA and MHGTMDA were evaluated using their models on our reconstructed dataset after removing data that could cause information leakage.

The results demonstrate that CoupleMDA consistently outperforms other baselines across different datasets, achieving approximately 0.5% improvement over the strongest baseline (NCNC). CoupleMDA exhibits superior performance on real-world datasets with well-structured graphs because it can better leverage the coupling advantages of structural and semantic features when target edges have comprehensive neighborhood connections. However, Zou’s dataset contains numerous isolated nodes where certain nodes have significantly more connections in specific categories than others, which obscures structural features and limits CoupleMDA’s potential. Despite this limitation, CoupleMDA still effectively captures semantic information and basic connectivity patterns, outperforming other state-of-the-art models. Crucially, methods that incorporate structural information during decoding (e.g., NCNC and CoupleMDA) surpass approaches that focus solely on encoding node embeddings. This observation indicates that considering structural features during decoding is essential, as processing node features in isolation may neglect edge-specific structural correlations.

### 4.5. Ablation Experiment

**Figure 3:**
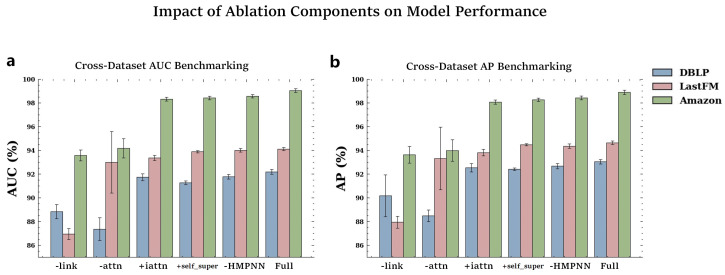
Impact of different components on CoupleMDA performance. Each bar represents performance of model variants: CoupleMDA-link (without common metapath guidance), CoupleMDA-attn (with batch-based attention), CoupleMDA+iattn (with inner attention aggregation), CoupleMDA-HMPNN (without pre-encoding), CoupleMDA+self_super (with self-supervision), and full CoupleMDA. Performance of the above model variants on datasets DBLP, LastFM and Amazon in terms of (**a**) AUC and (**b**) AP metrics.

To investigate the contributions of different components, we conducted ablation experiments on various CoupleMDA variants. Table 4 and Figure 3 present the ablation results of different variants on the DBLP, LastFM, and Amazon datasets. Table 5 shows the ablation results of different variants on Zou’s dataset. Model variants’ performance across training/validation/test sets on the Zou dataset (averaged over five trials) is detailed in Appendix A.2 (Table A11), with per-trial test-set TP/TN/FP/FN metrics in Table A12, Table A13, Table A14, Table A15, Table A16. Specifically, we designed the following variants:

- CoupleMDA-link: removes the co-metapath guided decoding mechanism that incorporates structural features.
- CoupleMDA-attn: replaces the inter-metapath self-attention with batch-based additive attention.
- CoupleMDA+iattn: substitutes the simple mean aggregation with intra-metapath attention.
- CoupleMDA+self_super: incorporates self-supervised learning for node-type and metapath-type classification.
- CoupleMDA-HMPNN: removes the HMPNN pre-encoding mechanism.

The results reveal performance degradation across all variants when specific components are removed or self-supervised learning is added. CoupleMDA’s superiority primarily stems from its co-metapath mechanism that couples semantic and structural information. The inter-metapath self-attention aggregation demonstrates more robust performance than batch-based additive attention, as it remains unaffected by batch configurations or sample ordering. Other components contribute marginally to the performance, while the addition of self-supervised learning fails to improve results. Although HMPNN provides limited enhancement to final prediction accuracy, it significantly accelerates model convergence during training.

### 4.6. Visualization Analysis

To investigate the internal mechanisms of our model, we visualize its intermediate results.

#### 4.6.1. Inter-Class Common Metapath Aggregation Weights

**Figure 4:**
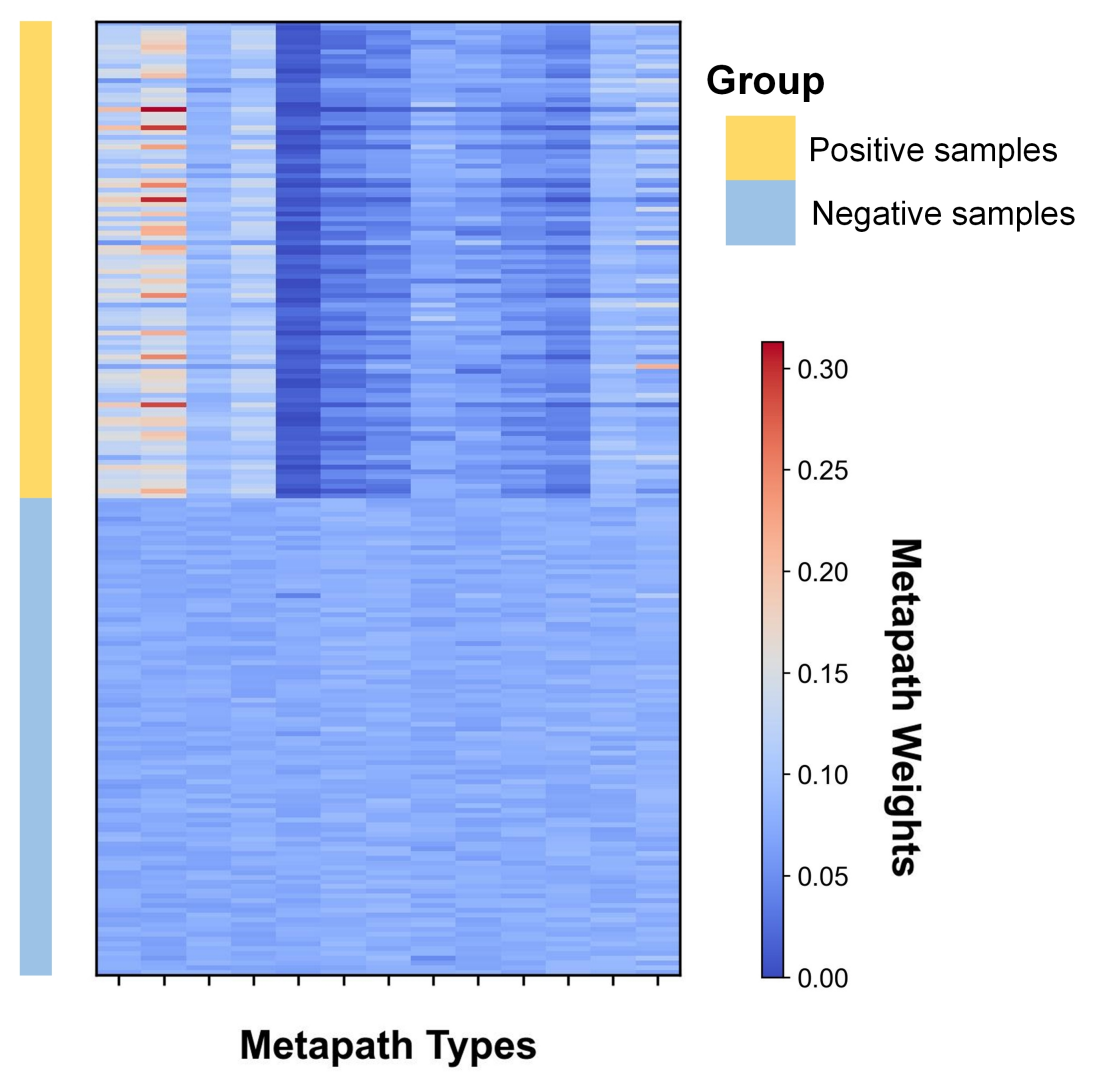
Attention Weight Distribution. Inter-class common metapath aggregation weight heatmaps for three datasets. Heatmap visualizing inter-class common metapath attention weights across three datasets. Each column represents a different metapath type; rows represent individual samples.

We extract the self-attention weights for inter-class common metapath aggregation, as shown in the heatmap of Figure 4. Each sample receives independent attention weights, where higher values indicate greater contribution of specific metapaths to model decisions. We group the weights for positive and negative samples separately.

For positive samples, the weights exhibit a skewed distribution, suggesting that model decisions primarily rely on specific common metapaths. Notably, the miRNA-mRNA-disease metapath provides the highest contribution, followed by miRNA-drug-disease and miRNA-circRNA-disease metapaths, while the miRNA-disease-miRNA-disease metapath shows minimal contribution. This pattern aligns with biological reality since the miRNA-mRNA-disease metapath contains richer connectivity in heterogeneous biological graphs. Our analysis indicates that effective common metapaths expand the receptive field of common neighbors, enabling the model to perceive broader topological structures without losing critical connection details.

In contrast, negative samples in Figure 4 display uniformly distributed weights, suggesting either insufficient structural information capture by the model or the absence of common metapaths for these unconnected node pairs, which consequently receive lower link prediction probabilities.

#### 4.6.2. Encoded Vector Visualization

**Figure 5:**
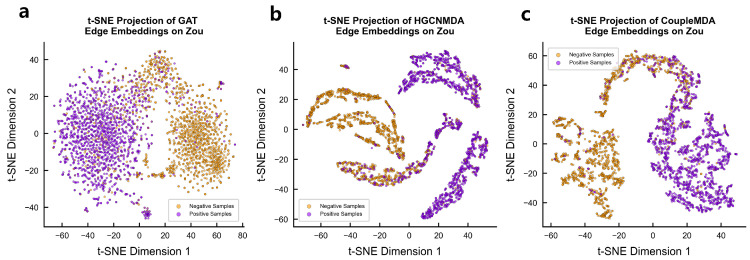
Node Embedding Visualization. t-SNE dimensionality reduction visualization comparing node embeddings from CoupleMDA (**c**) and baseline model GAT (**a**) and HGCNMDA (**b**) on Zou’s datasets.

We qualitatively evaluate the node representations learned by CoupleMDA against baseline models GAT and HGCNMDA through t-SNE visualization of their final-layer encoded vectors on Zou’s dataset. As shown in Figure 5, CoupleMDA-generated embeddings demonstrate significantly clearer cluster structures, exhibiting compact intra-class clustering and distinct inter-class boundaries in the 2D projection space. Different categories are separated by substantial margins, indicating superior discriminative power compared with the more overlapping distributions produced by baseline models.

### 4.7. Biomedical Case Study

To systematically evaluate the biomedical utility of CoupleMDA, we first performed differential expression analysis of miRNAs between prostate cancer (PCa) tissues and benign prostate tissues and then investigated the correlation between model predictions and differentially expressed miRNAs through statistical methods. Prostate cancer remains one of the most frequently diagnosed malignancies in males, where miRNAs serve as crucial biomarkers with specific expression profiles and therapeutic potential [75].

**Figure 6:**
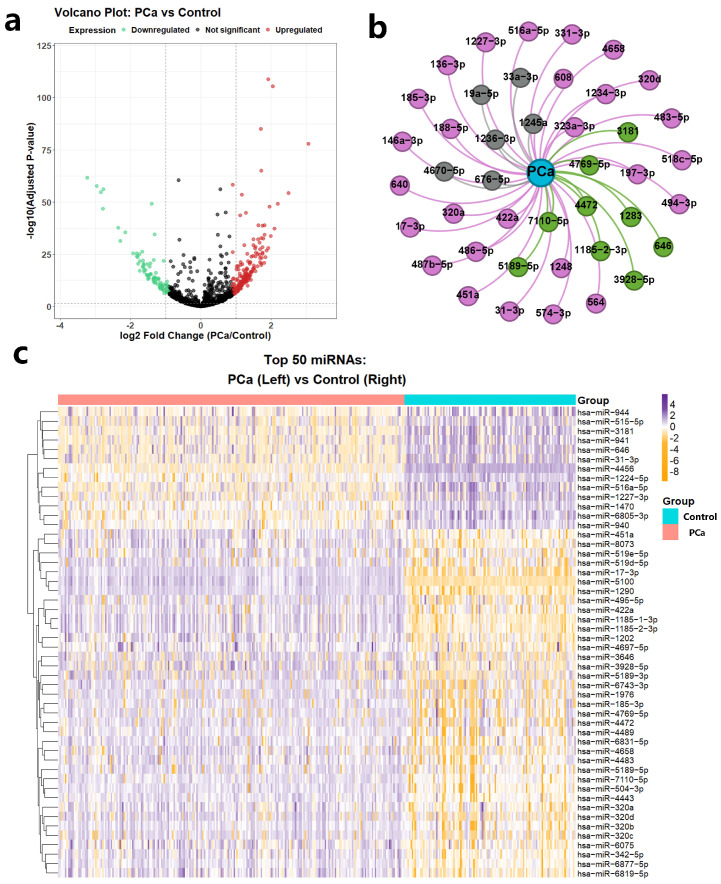
Case study of miRNA prediction related to PCa (**a**) Volcano plot of differential expression of all 1755 miRNAs, with red dots representing significantly upregulated RNAs and green dots representing significantly downregulated RNAs. (**b**) Validation results of the prediction in the dbDEMC database, where green nodes and edges indicate MDA verified in the dbDEMC database, red nodes represent RNAs considered significantly differentially expressed in the differential expression analysis of the GSE112264 dataset, and gray nodes and edges indicate MDA without experimental validation. All miRNA prefixes “hsa-miR” have been omitted. (**c**) Top 50 differentially expressed miRNAs in PCa samples. Orange blocks represent low-expression RNAs, and purple blocks represent high-expression RNAs.

The differential expression data were obtained from the GSE112264 dataset [76], containing 809 PCa tissue samples and 241 benign prostate tissue samples. Through volcano plot visualization, we identified 1755 differentially expressed miRNAs (Figure 6a), with the top 50 most significant expression shifts displayed in a heatmap (37 upregulated and 13 downregulated, Figure 6c). We selected PCa-associated miRNAs using established thresholds: |log_2_ FC| > 1 with adjusted *p*-value < 0.05. miRNAs meeting these criteria were classified as significantly differentially expressed, while others were labeled non-differentially expressed. This selection protocol integrated twofold expression changes and rigorous statistical validation (FDR < 5%) to ensure reliability.

CoupleMDA achieved prediction accuracies of AUC = 91% and AP = 85% when evaluating associations between all 1755 miRNAs and PCa. We validated the top 42 model-predicted PCa-related miRNAs against the dbDEMC database, as illustrated in Figure 6b. Among these, twenty-seven miRNAs received experimental support from existing literature (dbDEMC-EXP00020, EXP00032, EXP00042, EXP00044, EXP00045, EXP00046, EXP00403, EXP00469, EXP00639, EXP00640), while nine miRNAs overlapped with significantly differentially expressed RNAs identified in the GSE112264 analysis. This case study demonstrates the practical value and effectiveness of the proposed CoupleMDA method.

## 5. Methodology

This section provides a detailed description of the proposed CoupleMDA framework. The model represents a novel structure-semantics-coupled metapath aggregation approach for MDA link prediction in biological heterogeneous graphs. CoupleMDA consists of three core components: (1) metapath discovery, (2) an RGCN-based node pre-encoding module [69], and (3) a secondary encoding module based on metapath aggregation and a common metapath decoding module. Figure 1 illustrates these primary components and their workflow.

### 5.1. Overview of Model Framework

In link prediction, SF represented by common neighbors and path information are the core reference basis. The MPNN-then-SF architecture proposed by Wang et al. [48] adopts a two-stage design: first executing an MPNN on the original graph, then leveraging structural features to guide the pooling of MPNN-derived features. Inspired by this framework, our model generalizes the MPNN-then-SF approach from homogeneous to heterogeneous graphs, as illustrated in Figure 1.

Figure 1a displays the connection types in the constructed biological heterogeneous information network. Each category of biological entity contains multiple specific instance nodes, where each node connects to others according to the relationship types shown in Figure 1a. For example, the miRNA-type node “hsa-mir-122-5p” is linked to the mRNA-type node “nm_003045.5” in the database.

The workflow of CoupleMDA consists of four stages:

- Stage 1 (Figure 1b): Discover all metapath types of lengths 2 and 3 from the biological heterogeneous information network. For each metapath type, recursively traverse all nodes to identify all node-level instances and store them.
- Stage 2 (Figure 1c): learn node representations by aggregating multi-hop neighborhood features based on heterogeneous edge types in the original graph, enabling node embeddings to capture preliminary structural relationships.
- Stage 3 (Figure 1d): for each target node pair, generate two node-specific embeddings by aggregating their individual metapaths and one common metapath embedding by aggregating common metapaths between the pair.
- Stage 4 (Figure 1e): Input the Hadamard product of the two node embeddings and the weighted common metapath embedding into a decoder to produce the link prediction. Subsequent sections will elaborate on the core components of the model.

This framework offers two key advantages: First, the common metapath serves as a dominant structural feature to guide link prediction. Second, the pre-encoded node embeddings from the RGCN encapsulate preliminary connectivity and interaction patterns in the graph, thereby enhancing downstream structural feature embeddings. The strong coupling between RGCN and SF ensures high expressiveness for link prediction tasks. Furthermore, the RGCN-based pre-encoding addresses incomplete metapath issues caused by missing links [41].

### 5.2. MetaPath Discovery

The constructed biological heterogeneous graph comprises eight node types and sixteen edge types, as visually detailed in Figure 1a and Table 6. During data preprocessing, we identify all applicable metapaths for model learning. Prior to metapath discovery, the dataset is strictly partitioned: only miRNA-disease edges from the training set are retained in the original graph, while validation and test set edges are removed.

We define two metapath categories:

- Node-specific metapaths (P(v)): paths starting from target node *v* and ending with nodes of the same type (example: miRNA-circRNA-miRNA (MCM)).
- Common metapaths (P(v, u)): paths connecting heterogeneous node pairs (*v* and *u*), serving as bridges between different entity types (example: miRNA-lncRNA-mRNA-disease (MLRD)).

Node-specific metapaths (e.g., MCM) capture semantic interactions between homogeneous nodes, whereas common metapaths (e.g., MLRD) establish interpretable bridges between heterogeneous nodes and guide structural pooling.

We discovered 12 node-specific and 13 common metapaths (Figure 1b, Table 6). To enable self-attention mechanisms to autonomously learn metapath contributions, we exhaustively generate all permuted metapath combinations within specified lengths. According to γ-decay theory [26,77], higher-order structural features can be effectively approximated through low-order neighbors (small *h*-hops), as approximation errors decrease exponentially with *h*. Thus, metapaths with lengths ≤3 suffice to capture sufficient high-order structural information.

### 5.3. RGCN Node Pre-Encoding Module

Prior to feeding node vectors into RGCN, we apply type-specific linear transformations to project feature vectors of different node types into a common latent space. For each node $v^{T}$ of type *T*, the transformation is defined as

(6) $$h_{v}^{\left(0\right)}=h_{v}^{T}=W_{T}\cdot x_{v}^{T},$$

where $h_{v}^{\left(k\right)}$ denotes the feature representation of node *v* at layer *k*, $x_{v}^{T}\in R^{d_{T}}$ represents the original feature vector, and $W_{T}\in R^{d\times d_{T}}$ is the learnable weight matrix for type *T* nodes.

After a comprehensive evaluation, we select a single-layer RGCN as the pre-encoding module. This shallow architecture focuses on capturing direct first-order neighborhood features while preserving primitive topological information fidelity. As shown in Figure 1c, RGCN handles heterogeneous edges through relational-aware processing. The update rule for each node *v* in RGCN is formulated as

(7) $$\begin{array}{c} h_{v}^{\left(k+1\right)}=\sigma \left(W_{0}h_{v}^{\left(k\right)}+\sum_{r\in R}\sum_{u\in N_{r}\left(v\right)}\frac{1}{c_{r}}W_{r}h_{u}^{\left(k\right)}\right), \end{array}$$

where R denotes the set of all relation types, $N_{r}\left(v\right)$ represents the neighborhood of node *v* under relation *r*, and σ is the activation function.

### 5.4. MetaPath Full-Node Encoding

To comprehensively exploit semantic information from metapaths, we design a three-layer nested full-node encoder architecture that progressively processes information through the following stages: individual metapath encoding, intra-class metapath aggregation, and inter-class metapath aggregation.

For encoding individual metapaths, we employ linear transformation layers. The encoded representations of node-specific metapath P(v) and common metapath P(v,u) are formulated as

(8) $$\begin{array}{cc} h_{P(v)} & =W_{P}\cdot \mathrm{MEAN}\left(\left\{h_{t}|\forall t\in P\left(v\right)\right\}\right)\\ h_{P(v,u)} & =W_{P}\cdot \mathrm{MEAN}\left(\left\{h_{t}|\forall t\in P\left(v,u\right)\right\}\right), \end{array}$$

where metapaths of the same category share the identical weight matrix $W_{P}\in R^{d\times d_{T}}$.

After completing individual metapath encoding, we obtain encoded metapaths $M^{P}=\left\{h_{P_{1}},h_{P_{2}},\ldots ,h_{P_{m}}\right\}$ for category *P*. We then apply graph attention network [78] to compute weighted sums of all metapath encodings related to target nodes. The aggregated representation for category *P* is formulated as

(9) $$\begin{array}{cc} h^{P} & =\sigma \left(\sum_{h_{P_{i}}\in M^{P}}\alpha_{i}^{P}h_{P_{i}}\right)\\ \alpha_{i}^{P} & =\frac{\exp\left(\mathrm{LeakyReLU}(\Theta h_{P_{i}})\right)}{\sum_{s\in M^{P}}\exp\left(\mathrm{LeakyReLU}(\Theta s)\right)}, \end{array}$$

where $\Theta \in R^{d}$ denotes the learnable parameter.

As demonstrated by Yang et al. [25], neighbor attention mechanisms within homogeneous relations are non-essential, where simple mean aggregation achieves comparable effectiveness to attention-based approaches. We therefore formulate an alternative intra-class metapath aggregation as

(10) $$h^{P}=\sigma \left(\frac{1}{∥M^{P}∥}\sum_{h_{P_{i}}\in M^{P}}h_{P_{i}}\right).$$

Finally, we employ an inter-class metapath aggregation layer to integrate semantic information revealed by all metapaths. Unlike MAGNN’s batch-wise additive attention mechanism, we implement sample-wise multiplicative self-attention to aggregate metapath vectors. This design ensures batch independence while enabling adaptive attention weights per sample, thereby enhancing model robustness. Given encoded metapaths $H^{P}=\left\{h^{\left(P_{1}\right)},h^{\left(P_{2}\right)},\ldots ,h^{\left(P_{M}\right)}\right\}$, the inter-class aggregation is computed through

(11) $$\begin{array}{cc} X & =\tanh(W_{1}h^{P}+b_{1})\\ Q & =XW_{Q},\;K=XW_{K},\;V=XW_{V}\\ \beta & =W_{2}\left(\mathrm{softmax}\left(\frac{QK^{T}}{\sqrt{d_{k}}}\right)V\right)\\ h & =\sum_{i=1}^{M}\beta_{i}\cdot h_{v}^{\left(P_{i}\right)} \end{array}$$

where $X\in R^{d}$ denotes the activated output from the linear transformation of $h_{v}^{P}$, $Q,K,V\in R^{d_{k}}$ are linear projections of *X*, and $W_{2}\in R^{d_{k}\times 1}$ transforms attention weights $\beta \in R^{M}$ for all metapaths.

### 5.5. Link Prediction Decoder

For a specific link (u,v), we generate node-related metapaths P(u) and P(v), along with their common metapath P(u,v). Through HMPNN’s pre-encoding and full-node metapath encoding, we obtain encoded representations $h_{u}$, $h_{v}$, and $h_{uv}$ for both nodes and their link. The node encodings $h_{u}$ and $h_{v}$ are secondary encodings derived from pre-encoded features, which capture semantic interaction information between homogeneous nodes since the metapaths encoding these nodes connect homogeneous nodes at both ends. The link encoding $h_{uv}$ originates from common metapaths between nodes *u* and *v*, meaning these nodes are interconnected through intermediate nodes that form multiple communication pathways, constituting the most critical structural features of the connection.

**Figure 7:**
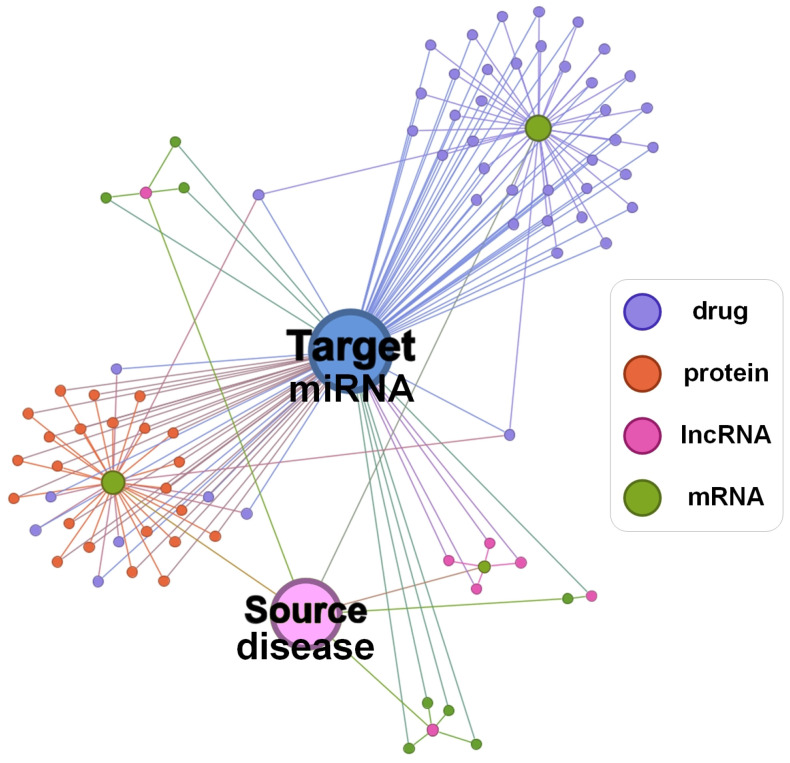
Simplified schematic diagram of the topological structure of the constructed heterogeneous graph of biological entities, with a specific disease as the source node and a specific miRNA as the target node.

We present the simplified topological structure schematic diagram of the previously constructed heterogeneous biological entity graph in Figure 7. This figure is drawn by selecting a specific disease as the source node and a specific miRNA as the target node and trimming most connections in the real dataset to retain only a few. Two potentially related nodes are connected through a pathway composed of multiple intermediate nodes. The source node connects to several hub nodes (e.g., mRNA-type nodes), while most satellite nodes linked to the target node associate with these hubs. All intermediate nodes provide structural insights for the source-target connection. Therefore, intermediate nodes along common metapaths serve as crucial structural features whose embeddings couple the link’s semantic features. This integration of structural and semantic features provides direct evidence for determining node connectivity. For node pairs with missing connections, we complete their metapaths using the nodes themselves, which aligns with biological intuition.

Based on the theoretical framework, we design a specialized decoder for link prediction:

(12) $$y=f\left(f_{1}\left(h_{u}⊙h_{v}\right)+\beta \cdot f_{2}\left(h_{uv}\right)\right)$$

where *f*, $f_{1}$, and $f_{2}$ denote multilayer perceptrons (MLPs) containing linear layers, layer normalization, and activation functions. The operator ⊙ represents the Hadamard product, and β is a learnable parameter. This decoder enhances link prediction performance by combining primary insights from node-level embeddings ($h_{u}⊙h_{v}$) with higher-order structural and semantic insights ($h_{uv}$).

## 6. Conclusions

This study addresses key limitations in current MDA prediction methodologies. First, by abandoning GIP similarity metrics and establishing strict data isolation, we resolve the persistent self-validation loop issue in prior research that artificially inflated performance metrics. Second, the proposed metapath-guided structural-semantic coupling mechanism simultaneously models multi-entity regulatory semantics and topological features, overcoming the information bottleneck in conventional GNN architectures. Third, our structure-aware decoding strategy enhances prediction accuracy and interpretability by dynamically weighting heterogeneous biological evidence through learnable metapath attention coefficients. Experimental validations across multiple scenarios demonstrate CoupleMDA’s robustness. The framework’s biological plausibility is confirmed through its ability to identify prostate cancer-associated miRNAs via drug-mediated pathways. These advancements not only establish new state-of-the-art performance in MDA prediction but also provide a blueprint for addressing feature-target coupling challenges in broader biomedical relation prediction tasks. Future work will extend this paradigm to multi-omics integration and temporal association modeling.

## Figures and Tables

**Table 1:** Data source of biological entity association data.

Association	Database	Association	Database
circleRNA-disease	Circ2Disease [56]	circRNA-disease	SomamiR [57]
disease-mRNA	DisGeNet [58]	disease-microbe	HMDAD [59]
drug-disease	SCMFDD [60]	drug-microbe	MDAD [61]
drug-mRNA	PharmGKB [62]	drug-protein	DrugBank [53]
lncRNA-disease	LncRNADisease [63]	lncRNA-miRNA	SNP [64]
lncRNA-mRNA	LncRNA2Target [65]	lncRNA-protein	NPInter [66]
miRNA-drug	SM2miR [67]	miRNA-mRNA	miRTarBase [68]
miRNA-protein	MHGTMDA [16]	mRNA-protein	MHGTMDA [16]

**Table 2:** Experiment results (%) on the DBLP, LastFM, and Amazon dataset for the link prediction task. The best performance for each metric in the table is highlighted in bold.

Models	DBLP		LastFM		Amazon	
	AUC	AP	AUC	AP	AUC	AP
RGCN	87.63 ± 0.14	89.92 ± 0.25	81.70 ± 0.39	86.27 ± 0.33	81.90 ± 0.54	77.89 ± 0.42
HGT	87.78 ± 0.23	89.14 ± 0.66	80.97 ± 0.54	83.41 ± 0.65	86.56 ± 4.07	84.12 ± 5.28
GATNE	75.63 ± 0.58	76.97 ± 0.37	86.62 ± 0.20	86.83 ± 0.21	96.58 ± 0.86	96.28 ± 0.41
HetGNN	84.31 ± 0.43	85.27 ± 0.59	88.16 ± 0.25	89.61 ± 0.32	95.71 ± 0.68	94.65 ± 0.82
Simple-HGN	87.89 ± 0.07	89.37 ± 0.08	84.73 ± 0.06	87.35 ± 0.05	97.57 ± 0.33	96.88 ± 0.46
HAN	88.15 ± 0.82	89.33 ± 1.17	88.16 ± 0.20	89.33 ± 0.18	97.16 ± 0.53	95.87 ± 0.74
MAGNN	89.76 ± 1.03	90.72 ± 1.12	88.89 ± 2.56	89.49 ± 1.75	98.43 ± 0.13	98.06 ± 0.21
NCNC	89.99 ± 1.44	91.81 ± 1.41	91.19 ± 0.34	91.56 ± 0.46	98.49 ± 0.19	98.35 ± 0.18
CoupleMDA	**92.19 ± 0.10**	**93.04 ± 0.09**	**94.11 ± 0.13**	**94.64 ± 0.15**	**99.04 ± 0.16**	**98.89 ± 0.18**

**Table 3:** Experiment results (%) on the test set of Zou’s dataset for the MDA link prediction task. The best performance for each metric in the table is highlighted in bold.

Model	AUC	AP	Precision	Recall	F1 Score
GATNE	75.70 ± 0.64	77.32 ± 1.25	56.31 ± 1.37	**99.66 ± 0.33**	71.96 ± 1.36
HetGNN	90.06 ± 0.35	89.60 ± 0.58	84.21 ± 0.59	78.46 ± 1.08	81.24 ± 0.17
Simple-HGN	94.16 ± 0.86	93.02 ± 1.07	82.10 ± 2.57	94.32 ± 1.19	87.79 ± 1.99
HAN	93.95 ± 0.97	92.15 ± 0.38	86.71 ± 1.44	88.25 ± 0.51	87.48 ± 0.39
MAGNN	94.63 ± 0.67	93.97 ± 0.41	86.78 ± 1.09	91.79 ± 0.72	89.21 ± 1.33
NCNC	94.96 ± 0.55	94.42 ± 0.42	85.47 ± 1.05	94.62 ± 1.21	89.30 ± 1.27
HGCNMDA	93.92 ± 0.18	92.71 ± 0.58	89.64 ± 0.18	88.35 ± 0.76	88.99 ± 0.31
MHGTMDA	94.64 ± 0.31	93.51 ± 0.77	88.63 ± 0.43	89.97 ± 1.39	89.29 ± 0.56
CoupleMDA	**95.36 ± 0.35**	**94.84 ± 0.32**	**90.51 ± 1.69**	91.92 ± 1.55	**90.21 ± 0.58**

**Table 4:** Quantitative results of ablation study (%) on the test set of DBLP, LastFM, and Amazon datasets. The best performance for each metric in the table is highlighted in bold.

Variant	DBLP		LastFM		Amazon	
	AUC	AP	AUC	AP	AUC	AP
CoupleMDA-link	88.84 ± 0.60	90.18 ± 1.76	86.95 ± 0.45	87.95 ± 0.50	93.57 ± 0.46	93.63 ± 0.71
CoupleMDA-attn	87.36 ± 0.96	88.48 ± 0.49	93.00 ± 2.59	93.32 ± 2.63	94.18 ± 0.81	93.99 ± 0.90
CoupleMDA+iattn	91.74 ± 0.29	92.54 ± 0.35	93.36 ± 0.21	93.81 ± 0.27	98.31 ± 0.16	98.07 ± 0.17
CoupleMDA-HMPNN	91.27 ± 0.16	92.41 ± 0.11	93.89 ± 0.10	94.48 ± 0.09	98.42 ± 0.15	98.27 ± 0.14
CoupleMDA+self_super	91.78 ± 0.19	92.67 ± 0.22	94.00 ± 0.15	94.36 ± 0.19	98.57 ± 0.14	98.42 ± 0.16
CoupleMDA	**92.19 ± 0.20**	**93.04 ± 0.19**	**94.11 ± 0.13**	**94.64 ± 0.15**	**99.04 ± 0.16**	**98.89 ± 0.18**

**Table 5:** Quantitative results of ablation study (%) on the test set of Zou’s dataset. The best performance for each metric in the table is highlighted in bold.

Variant	AUC	AP	Precision	Recall	F1 Score
CoupleMDA-link	94.84 ± 0.59	94.31 ± 0.31	84.70 ± 0.40	**94.16 ± 0.51**	89.18 ± 0.96
CoupleMDA-attn	94.77 ± 0.72	94.21 ± 0.79	87.03 ± 1.06	91.66 ± 0.56	89.29 ± 1.34
CoupleMDA+iattn	95.16 ± 0.47	94.64 ± 0.42	86.90 ± 1.07	93.92 ± 0.69	89.34 ± 0.46
CoupleMDA+self_super	94.82 ± 0.75	94.57 ± 0.09	87.17 ± 1.15	92.32 ± 0.85	89.67 ± 1.28
CoupleMDA-HMPNN	94.96 ± 0.57	94.20 ± 0.68	86.49 ± 1.18	91.66 ± 0.31	89.00 ± 0.25
CoupleMDA	**95.35 ± 0.45**	**94.84 ± 0.32**	**90.51 ± 1.69**	91.92 ± 1.55	**90.21 ± 0.58**

**Table 6:** Twelve node-specific metapaths and thirteen common metapaths discovered from heterogeneous graphs.

Node-Specific MetaPaths	Common MetaPaths
miRNA–mRNA–miRNA	miRNA–drug–disease
miRNA–drug–miRNA	miRNA–mRNA–disease
miRNA–circRNA–miRNA	miRNA–lncRNA–disease
miRNA–lncRNA–miRNA	miRNA–circRNA–disease
miRNA–protein–miRNA	miRNA–disease–miRNA–disease
miRNA–disease–miRNA	miRNA–drug–mRNA–disease
disease–mRNA–disease	miRNA–drug–microbe–disease
disease–drug–disease	miRNA–mRNA–drug–disease
disease–circRNA–disease	miRNA–mRNA–lncRNA–disease
disease–lncRNA–disease	miRNA–protein–drug–disease
disease–microbe–disease	miRNA–protein–mRNA–disease
disease–miRNA–disease	miRNA–protein–lncRNA–disease
	miRNA–lncRNA–mRNA–disease

## Data Availability

The source code and data of this study are available at https://github.com/lizhj39/CoupleMDA (accessed on 17 March 2025).

## Appendix A

### Appendix A.1

**Table A1:** Comparative performance analysis of heterogeneous graph neural networks (mean ± std%). All metrics were averaged over five independent runs. AUC (area under curve), AP (average precision), and Acc (accuracy).

Model	Subdataset	Performance Metrics					
		AUC	AP	Precision	Recall	F1 Score	Acc
GATNE	Train	76.84 ± 0.62	78.47 ± 1.23	57.53 ± 1.32	99.33 ± 0.31	73.18 ± 1.34	59.47 ± 1.03
	Val	76.12 ± 0.57	77.82 ± 1.17	56.79 ± 1.26	99.78 ± 0.29	72.51 ± 1.23	58.83 ± 0.92
	Test	75.70 ± 0.64	77.32 ± 1.25	56.31 ± 1.37	99.66 ± 0.33	71.96 ± 1.36	61.35 ± 0.51
HetGNN	Train	91.23 ± 0.32	90.78 ± 0.51	85.52 ± 0.57	79.82 ± 1.02	82.53 ± 0.16	83.03 ± 0.82
	Val	90.53 ± 0.34	90.14 ± 0.56	84.63 ± 0.59	79.23 ± 1.09	81.79 ± 0.17	82.32 ± 0.73
	Test	90.06 ± 0.35	89.60 ± 0.58	84.21 ± 0.59	78.46 ± 1.08	81.24 ± 0.17	81.88 ± 0.60
Simple- HGN	Train	95.38 ± 0.81	94.17 ± 1.03	83.48 ± 2.52	95.47 ± 1.12	88.97 ± 1.92	89.82 ± 0.91
	Val	94.62 ± 0.83	93.53 ± 1.06	82.57 ± 2.46	95.83 ± 1.21	88.31 ± 1.86	89.13 ± 0.81
	Test	94.16 ± 0.86	93.02 ± 1.07	82.10 ± 2.57	94.32 ± 1.19	87.79 ± 1.99	87.48 ± 0.77
HAN	Train	95.21 ± 0.91	93.48 ± 0.36	88.03 ± 1.36	89.53 ± 0.46	88.82 ± 0.36	88.63 ± 0.61
	Val	94.43 ± 0.87	92.81 ± 0.41	87.24 ± 1.31	89.81 ± 0.52	87.93 ± 0.40	87.82 ± 0.51
	Test	93.95 ± 0.97	92.15 ± 0.38	86.71 ± 1.44	88.25 ± 0.51	87.48 ± 0.39	87.02 ± 0.94
MAGNN	Train	95.79 ± 0.63	95.19 ± 0.39	88.12 ± 1.03	93.04 ± 0.66	90.51 ± 1.27	89.84 ± 0.72
	Val	95.13 ± 0.61	94.52 ± 0.43	87.31 ± 1.06	92.51 ± 0.71	89.72 ± 1.31	89.15 ± 0.61
	Test	94.63 ± 0.67	93.97 ± 0.41	86.78 ± 1.09	91.79 ± 0.72	89.21 ± 1.33	89.24 ± 0.59
NCNC	Train	96.27 ± 0.52	95.72 ± 0.39	86.83 ± 1.01	96.03 ± 1.16	90.62 ± 1.21	90.29 ± 0.62
	Val	95.53 ± 0.53	95.04 ± 0.41	86.05 ± 1.06	95.84 ± 1.22	90.04 ± 1.26	89.63 ± 0.52
	Test	94.96 ± 0.55	94.42 ± 0.42	85.47 ± 1.05	94.62 ± 1.21	89.30 ± 1.27	89.28 ± 0.23
HGCNMDA	Train	95.12 ± 0.16	94.03 ± 0.56	90.82 ± 0.16	89.83 ± 0.71	89.51 ± 0.29	89.53 ± 0.51
	Val	94.31 ± 0.19	93.32 ± 0.59	90.13 ± 0.19	90.21 ± 0.76	89.32 ± 0.31	89.22 ± 0.41
	Test	93.92 ± 0.18	92.71 ± 0.58	89.64 ± 0.18	88.35 ± 0.76	88.99 ± 0.31	88.99 ± 0.20
MHGTMDA	Train	95.88 ± 0.29	94.81 ± 0.71	89.93 ± 0.41	91.53 ± 1.31	90.63 ± 0.51	90.03 ± 0.52
	Val	95.21 ± 0.31	94.13 ± 0.76	89.14 ± 0.43	91.04 ± 1.36	89.81 ± 0.57	89.34 ± 0.41
	Test	94.64 ± 0.31	93.51 ± 0.77	88.63 ± 0.43	89.97 ± 1.39	89.29 ± 0.56	88.71 ± 0.30
CoupleMDA	Train	96.13 ± 0.31	95.63 ± 0.29	91.82 ± 1.61	93.52 ± 1.51	91.53 ± 0.56	91.23 ± 0.51
	Val	95.72 ± 0.33	95.21 ± 0.31	91.03 ± 1.66	92.83 ± 1.56	90.72 ± 0.59	90.42 ± 0.41
	Test	95.36 ± 0.35	94.84 ± 0.32	90.51 ± 1.69	91.92 ± 1.55	90.21 ± 0.58	90.64 ± 0.69

**Table A2:** Experimental results of GATNE model on different runs in the test set. (TP: true positives, TN: true negatives, FP: false positives, FN: false negatives).

Exp	TP	TN	FP	FN	Precision (%)	Recall (%)	F1 Score (%)	Acc (%)
1	1513	352	1171	10	56.37	99.34	71.93	61.23
2	1522	339	1184	1	56.25	99.93	71.98	61.10
3	1515	369	1154	8	56.76	99.47	72.28	61.85
4	1518	330	1193	5	55.99	99.67	71.71	60.67
5	1520	365	1158	3	56.76	99.80	72.36	61.88

**Table A3:** Experimental results of HetGNN model on different runs in the test set.

Exp	TP	TN	FP	FN	Precision (%)	Recall (%)	F1 Score (%)	Acc (%)
1	1184	1315	208	339	85.06	77.74	81.23	82.04
2	1195	1299	224	328	84.21	78.46	81.24	81.88
3	1189	1289	234	334	83.56	78.07	80.72	81.35
4	1193	1302	221	330	84.37	78.33	81.24	81.91
5	1206	1280	243	317	83.23	79.19	81.16	81.62

**Table A4:** Experimental results of Simple-HGN model on different runs in the test set.

Exp	TP	TN	FP	FN	Precision (%)	Recall (%)	F1 Score (%)	Acc (%)
1	1422	1257	266	101	84.24	93.37	88.57	87.95
2	1454	1225	298	69	82.99	95.47	88.79	87.95
3	1402	1284	239	121	85.44	92.06	88.62	88.18
4	1438	1197	326	85	81.52	94.42	87.50	86.51
5	1431	1213	310	92	82.19	93.96	87.68	86.80

**Table A5:** Experimental results of HAN model on different runs in the test set.

Exp	TP	TN	FP	FN	Precision (%)	Recall (%)	F1 Score (%)	Acc (%)
1	1344	1317	206	179	86.71	88.25	87.47	87.36
2	1337	1278	245	186	84.51	87.79	86.12	85.85
3	1360	1324	199	163	87.24	89.30	88.25	88.12
4	1324	1341	182	199	87.92	86.93	87.42	87.49
5	1329	1297	226	194	85.47	87.26	86.35	86.21

**Table A6:** Experimental results of MAGNN model on different runs in the test set.

Exp	TP	TN	FP	FN	Precision (%)	Recall (%)	F1 Score (%)	Acc (%)
1	1427	1313	210	96	87.17	93.70	90.32	89.95
2	1398	1328	195	125	87.76	91.79	89.73	89.49
3	1392	1300	223	131	86.19	91.40	88.72	88.38
4	1423	1287	236	100	85.77	93.43	89.44	88.97
5	1383	1341	182	140	88.37	90.81	89.57	89.43

**Table A7:** Experimental results of NCNC model on different runs in the test set.

Exp	TP	TN	FP	FN	Precision (%)	Recall (%)	F1 Score (%)	Acc (%)
1	1449	1274	249	74	85.34	95.14	89.97	89.40
2	1423	1292	231	100	86.03	93.43	89.58	89.13
3	1468	1245	278	55	84.08	96.39	89.81	89.07
4	1419	1311	212	104	87.00	93.17	89.98	89.63
5	1434	1282	241	89	85.61	94.16	89.68	89.17

**Table A8:** Experimental results of HGCNMDA model on different runs in the test set.

Exp	TP	TN	FP	FN	Precision (%)	Recall (%)	F1 Score (%)	Acc (%)
1	1344	1368	155	179	89.66	88.25	88.95	89.03
2	1346	1365	158	177	89.49	88.38	88.93	89.00
3	1343	1366	157	180	89.53	88.18	88.85	88.94
4	1349	1366	157	174	89.58	88.58	89.07	89.13
5	1342	1364	159	181	89.41	88.12	88.76	88.84

**Table A9:** Experimental results of MHGTMDA model on different runs in the test set.

Exp	TP	TN	FP	FN	Precision (%)	Recall (%)	F1 Score (%)	Acc (%)
1	1370	1349	174	153	88.73	89.95	89.34	89.26
2	1375	1349	174	148	88.77	90.28	89.52	89.43
3	1378	1344	179	145	88.50	90.48	89.48	89.36
4	1374	1347	176	149	88.65	90.22	89.42	89.33
5	1360	1349	174	163	88.66	89.30	88.98	88.94

**Table A10:** Experimental results of CoupleMDA model on different runs in the test set.

Exp	TP	TN	FP	FN	Precision (%)	Recall (%)	F1 Score (%)	Acc (%)
1	1398	1356	167	125	89.33	91.79	90.54	90.41
2	1415	1336	187	108	88.33	92.91	90.56	90.32
3	1413	1379	144	110	90.75	92.78	91.75	91.66
4	1383	1388	135	140	91.11	90.81	90.96	90.97
5	1369	1368	155	154	89.83	89.89	89.86	89.86

### Appendix A.2

**Table A11:** Performance comparison of CoupleMDA variants with different architectures (mean ± std%). Performance metrics are reported as mean ± standard deviation over five runs. AUC (area under curve), AP (average precision), Acc (accuracy).

Model Variant	Subdataset	Performance Metrics					
		AUC	AP	Precision	Recall	F1 Score	Acc
CoupleMDA- link	Train	95.12 ± 0.38	94.72 ± 0.28	86.34 ± 0.42	91.85 ± 0.55	89.01 ± 0.42	89.12 ± 0.62
	Val	95.47 ± 0.35	95.02 ± 0.30	85.83 ± 0.40	94.28 ± 0.50	89.83 ± 0.38	89.55 ± 0.58
	Test	94.84 ± 0.59	94.31 ± 0.31	84.70 ± 0.40	94.16 ± 0.51	89.18 ± 0.96	88.15 ± 1.10
CoupleMDA- attn	Train	94.68 ± 0.65	94.15 ± 0.72	87.12 ± 1.08	91.23 ± 0.58	89.08 ± 1.25	88.92 ± 0.82
	Val	95.03 ± 0.62	94.67 ± 0.68	86.95 ± 1.05	93.88 ± 0.55	89.73 ± 1.20	89.35 ± 0.78
	Test	94.77 ± 0.72	94.21 ± 0.79	87.03 ± 1.06	91.66 ± 0.56	89.29 ± 1.34	89.03 ± 0.59
CoupleMDA +iattn	Train	95.23 ± 0.41	94.82 ± 0.38	87.45 ± 1.05	92.98 ± 0.65	89.82 ± 0.50	89.34 ± 0.70
	Val	95.63 ± 0.38	95.24 ± 0.35	87.02 ± 1.02	94.42 ± 0.62	90.32 ± 0.47	89.92 ± 0.65
	Test	95.16 ± 0.47	94.64 ± 0.42	86.90 ± 1.07	93.92 ± 0.69	89.34 ± 0.46	89.27 ± 0.60
CoupleMDA- iattn+ self_super	Train	94.85 ± 0.68	94.63 ± 0.12	87.52 ± 1.15	92.05 ± 0.80	89.58 ± 1.22	88.95 ± 0.75
	Val	95.22 ± 0.64	95.01 ± 0.10	87.25 ± 1.12	93.85 ± 0.78	90.12 ± 1.18	89.62 ± 0.72
	Test	94.82 ± 0.75	94.57 ± 0.09	87.17 ± 1.15	92.32 ± 0.85	89.67 ± 1.28	89.49 ± 0.70
CoupleMDA- HMPNN	Train	94.93 ± 0.52	94.42 ± 0.60	86.85 ± 1.18	91.28 ± 0.33	88.92 ± 0.28	88.45 ± 0.55
	Val	95.31 ± 0.49	94.88 ± 0.56	86.63 ± 1.15	92.84 ± 0.30	89.45 ± 0.25	89.12 ± 0.52
	Test	94.96 ± 0.57	94.20 ± 0.68	86.49 ± 1.18	91.66 ± 0.31	89.00 ± 0.25	88.58 ± 0.50
CoupleMDA	Train	95.47 ± 0.30	94.91 ± 0.25	87.19 ± 1.65	92.11 ± 1.50	89.58 ± 0.55	89.29 ± 0.55
	Val	96.05 ± 0.28	95.33 ± 0.27	86.95 ± 1.60	94.22 ± 1.48	90.35 ± 0.53	89.38 ± 0.52
	Test	95.35 ± 0.45	94.84 ± 0.32	90.51 ± 1.69	91.92 ± 1.55	90.21 ± 0.58	89.93 ± 0.30

**Table A12:** Experimental results of CoupleMDA-link variant on different runs in the test set.

Exp	TP	TN	FP	FN	Precision (%)	Recall (%)	F1 Score (%)	Acc (%)
1	1419	1259	264	104	84.31	93.17	88.52	87.92
2	1434	1270	253	89	85.00	94.16	89.35	88.77
3	1445	1283	240	78	85.76	94.88	90.09	89.56
4	1410	1228	295	113	82.70	92.58	87.36	86.61
5	1423	1255	268	100	84.15	93.43	88.55	87.92

**Table A13:** Experimental results of CoupleMDA-attn variant on different runs in the test set.

Exp	TP	TN	FP	FN	Precision (%)	Recall (%)	F1 Score (%)	Acc (%)
1	1387	1297	226	136	85.99	91.07	88.46	88.12
2	1393	1315	208	130	87.01	91.46	89.18	88.90
3	1397	1323	200	126	87.48	91.73	89.55	89.30
4	1401	1332	191	122	88.00	91.99	89.95	89.72
5	1401	1314	209	122	87.02	91.99	89.44	89.13

**Table A14:** Experimental results of CoupleMDA+iattn variant on different runs in the test set.

Exp	TP	TN	FP	FN	Precision (%)	Recall (%)	F1 Score (%)	Acc (%)
1	1403	1309	214	120	86.77	92.12	89.36	89.03
2	1431	1324	199	92	87.79	93.96	90.77	90.45
3	1388	1304	219	135	86.37	91.14	88.69	88.38
4	1417	1334	189	106	88.23	93.04	90.57	90.32
5	1391	1295	228	132	85.92	91.33	88.54	88.18

**Table A15:** Experimental results of CoupleMDA+self_super variant on different runs in the test set.

Exp	TP	TN	FP	FN	Precision (%)	Recall (%)	F1 Score (%)	Acc (%)
1	1411	1316	207	112	87.21	92.65	89.84	89.53
2	1409	1328	195	114	87.84	92.51	90.12	89.86
3	1410	1304	219	113	86.56	92.58	89.47	89.10
4	1405	1341	182	118	88.53	92.25	90.35	90.15
5	1415	1290	233	108	85.86	92.91	89.25	88.80

**Table A16:** Experimental results of CoupleMDA-HMPNN variant on different runs in the test set.

Exp	TP	TN	FP	FN	Precision (%)	Recall (%)	F1 Score (%)	Acc (%)
1	1396	1305	218	127	86.49	91.66	89.00	88.67
2	1395	1314	209	128	86.97	91.60	89.22	88.94
3	1391	1284	239	132	85.34	91.33	88.23	87.82
4	1400	1299	224	123	86.21	91.92	88.97	88.61
5	1396	1308	215	127	86.65	91.66	89.09	88.77
